# Multidrug-Resistant Candida in Bloodstream Infections: A Growing Concern in Indian Healthcare

**DOI:** 10.7759/cureus.85420

**Published:** 2025-06-05

**Authors:** Shailendra K Yadav, D Himanshu, Prashant Gupta, Anuradha Nischal, Virendra Atam, Wahid Ali, Ambuj Yadav, Vijay Sonkar, Vivek Kumar

**Affiliations:** 1 Department of Medicine, King George's Medical University, Lucknow, IND; 2 Department of Microbiology, King George's Medical University, Lucknow, IND; 3 Department of Pharmacology, King George's Medical University, Lucknow, IND; 4 Department of Medicine, King George’s Medical University, Lucknow, IND; 5 Department of Pathology, King George's Medical University, Lucknow, IND

**Keywords:** bloodstream infection, candida, echinocandin, multidrug resistance, susceptibility

## Abstract

Background and introduction: Bloodstream Candida infection, or candidemia, is a serious fungal infection caused by Candida species, often occurring in hospitalized or immunocompromised patients. It can lead to severe complications, including sepsis and multi-organ failure. The emergence of antifungal resistance, particularly in species like Candida auris and Candida glabrata, poses a significant challenge to treatment.

Material and methods: This was a hospital-based retrospective study conducted over a five-year period, from January 2020 to December 2024. All bloodstream infection clinical sample details were collected from the microbiology laboratory with Candida infection. A total of 1058 isolates of Candida species were included in the study. The study samples were the clinically relevant Candida isolates for which antifungal susceptibility testing was done at a tertiary care hospital in North India. The data were entered into a database, and an antibiogram for Candida species was generated.

Results: Candida tropicalis was the most prevalent, with 340 isolates, which make up 32.13% of the total isolates of Candida. Candida parapsilosis was the next most common, with 145 isolates, comprising 13.7% of the total isolates. Candida auris accounted for 100 isolates, representing 9.45% of the total isolates. Candida albicans, the fourth most common Candida genus, was 96 isolates (9.07%), making it less common in comparison to Candida non-albicans. Isolates from paediatric patients dominate the list, with significantly higher samples compared to the others, followed by adult patients from medicine departments with 218 samples, showing its importance in overall sample contributions. Among antifungals, caspofungin had the highest sensitivity across most species, e.g., Candida albicans at 94.7%, Candida parapsilosis at 98.6%, and Candida tropicalis at 97.5%. Caspofungin was found to be 96% sensitive to Candida auris. Fluconazole displays variable effectiveness as low susceptibility was found in Candida auris (8.9%) and Candida glabrata (35.1%). Voriconazole maintains relatively high susceptibility, with better performance compared to fluconazole in most species, e.g., Candida albicans (84%) and Candida glabrata (78.9%).

Conclusion: Candida tropicalis emerged as the most prevalent species, followed by Candida parapsilosis, Candida auris, and Candida albicans, with non-albicans species being more common overall. Candida resistance is a growing clinical challenge that requires urgent attention.

At one time, Candida albicans was the most common organism; however, Candida auris has now emerged as the most common resistant pathogen in hospitals across India, with its resistance continuing to increase.

## Introduction

Candida infection, caused by Candida species, is a common fungal infection that can affect various parts of the body, including the skin, mucous membranes, and internal organs. The most frequent species involved in these infections is Candida albicans, although non-albicans species like Candida auris and Candida tropicalis have also been increasingly identified in clinical settings [1]. Candida infections typically occur when there is an imbalance in the body's normal flora, often due to factors such as antibiotic use, immunosuppression, diabetes, and hormonal changes. Symptoms vary depending on the location and severity of the infection, ranging from mild oral thrush to more severe systemic infections like candidemia [2,3]. Diagnosis is often confirmed through clinical examination, microbiological cultures, and molecular techniques. Invasive candidiasis often presents with fever and chills; this form can affect internal organs and is particularly dangerous for hospitalized patients [4-6]. Treatment involves antifungal medications, with the choice of therapy depending on the site and severity of the infection [7]. Resistance to common antifungal drugs is a growing concern, highlighting the need for continuous research into new therapeutic strategies. Candida infections are a leading cause of hospital-acquired infections (HAIs) in India, with Candida albicans being the most common species isolated. However, non-albicans species such as Candida tropicalis, Candida glabrata, and Candida krusei are also increasingly identified, often showing resistance to common antifungal treatments. A multicenter study conducted across India found that approximately 10-15% of bloodstream infections in ICU patients were due to Candida species, with the mortality rate varying between 30-60% depending on the presence of underlying conditions like diabetes, cancer, or HIV/AIDS [8].

Diabetes mellitus is a significant risk factor for candidiasis in India, where its prevalence is rising rapidly. People with diabetes are more susceptible to both superficial and systemic Candida infections, particularly Candida albicans in the skin and mucosal membranes, and Candida glabrata in more serious bloodstream infections. The increasing use of immunosuppressive drugs and organ transplant procedures also contributes to the rise of Candida infections in both hospitalized and outpatient populations. The mortality rate associated with Candida infections in India is alarming, particularly in critically ill patients. A study published in the Indian Journal of Medical Research reported a 50-60% mortality rate among patients with candidemia in intensive care units, with higher rates observed in those who had underlying conditions such as diabetes, renal failure, or cancer [9]. Mortality is often linked to delayed diagnosis and inadequate antifungal therapy, with many infections going undetected due to the lack of rapid diagnostic tools and expertise in identifying invasive candidiasis. The challenges of diagnosing and treating Candida infections are compounded by the growing resistance to common antifungal agents, particularly azoles. Resistance to fluconazole, one of the most widely used antifungal drugs in India, has been reported in both Candida albicans and non-albicans species, further complicating treatment outcomes [10]. This highlights the need for improved diagnostic techniques, targeted antifungal therapy, and enhanced surveillance systems to monitor antifungal resistance. Candida infections, particularly invasive forms like candidemia, have emerged as a significant cause of morbidity and mortality, especially in immunocompromised patients. One of the primary concerns in the treatment of Candida infections is the growing problem of antifungal resistance. Resistance has been particularly noted in species such as Candida glabrata, Candida tropicalis, and Candida krusei, with Candida albicans also showing increasing resistance to some classes of antifungals [11]. Resistance mechanisms include altered drug targets, efflux pump overexpression, biofilm formation, and mutations that prevent drug binding, complicating the effective treatment of these infections.

This study aimed to analyze the various Candida isolates obtained from blood cultures in a tertiary care hospital between 2020 and 2024, along with their antifungal susceptibility patterns. The emergence of resistance not only reduces the efficacy of commonly used antifungals like fluconazole but also necessitates the use of more expensive or toxic alternatives, such as echinocandins or amphotericin B, which may not always be effective against resistant strains [12]. Candida infections are associated with high mortality rates, particularly when the infection becomes invasive. The mortality rate in patients with candidemia ranges from 20% to 50%, depending on factors such as the patient’s underlying conditions, the timeliness of antifungal therapy, and the presence of antifungal resistance [13]. Early diagnosis and prompt treatment are crucial in improving survival outcomes, but the rising incidence of drug-resistant Candida strains further complicates treatment regimens and may lead to worse prognoses [14]. Studies have shown that delayed appropriate antifungal therapy is associated with increased mortality, highlighting the importance of rapid identification of the causative organism and susceptibility testing. As such, strategies to combat resistance, such as stewardship programs, antifungal susceptibility testing, and the development of new antifungal agents, are essential in reducing both the prevalence and the mortality associated with Candida infections [15].

## Materials and methods

This is a retrospective study of a five-year duration from January 2020 to December 2024. Data was collected from the microbiology department of King George’s Medical University, Lucknow, a tertiary care hospital of 4000 beds in northern India. This study included data from 1058 positive blood samples. Blood samples were collected in blood culture bottles and processed in the automated blood culture system BacT/Alert microbial identification system. The positive samples were processed for culture on sheep blood agar medium and incubated for 24 hours at 37°C. After that, organisms were identified by matrix-assisted laser desorption ionization time-of-flight mass spectrometry (MALDI-TOF MS; Vitek MS, Biomerieux, Marcy-l'Étoile, France). Further samples were tested for antimicrobial susceptibility testing (AST) by the disk diffusion method and minimum inhibitory concentration through the broth dilution method. 

All the procedures were performed as per Clinical and Laboratory Standards Institute (CLSI M27M44S) guidelines. Sample distribution of isolates was studied. The data collected was entered into Excel (Microsoft, Redmond, WA, USA) to generate an antibiogram for Candida. Candida species tested include Candida albicans, Candida auris, Candida glabrata, Candida krusei, Candida parapsilosis, Candida tropicalis and Candida utilis. Antifungal agents evaluated were caspofungin, fluconazole, and voriconazole. For each Candida species, the number of isolates tested and their respective susceptibility rates (percentages) to the antifungals are provided.

Clinical characteristics of samples were expressed as numbers and percentages for categorical variables. The association of these variables with C. species was analyzed using the χ2 test. Comparison of antifungal susceptibility between different C. species was inferred using the χ2 test results. All analyses were conducted using SPSS version 24.0 software (IBM Corp., Armonk, NY, USA). A P value < 0.05 was considered statistically significant. Approval for this study was obtained from our institutional ethical committee at King George's Medical University, Lucknow (no.115thECM IIA/P29).

## Results

Samples received every year showed that the infection was increasing year by year. A moderate positive relationship is indicated in Table 1, meaning as the year increased, the sample size tended to increase slightly, though the relationship was not very strong. As per Table 2, Paediatrics dominated the list, with significantly higher samples compared to the others. Medicine followed with 218 samples, showed its importance in overall sample contributions as per Table 2. Other departments, such as Gynaecology, General Surgery, and the Trauma unit, had moderate sample counts, indicating balanced contributions. The remaining departments in the top 10 contributed between 26 and 42 samples, primarily focusing on specialized care.

**Table 1:** Sample distribution according to year

Year	Samples
2020	137
2021	246
2022	261
2023	162
2024	252

**Table 2:** Department-wise sample distribution

DEPARTMENT	SAMPLES	DEPARTMENT	SAMPLES
Pediatrics	343	Hematology	08
Medicine	218	Ophthalmology	07
Gynecology	70	Physical medicine	06
General Surgery	60	Cardiology	04
Trauma unit	59	Trauma & Emergency	04
Surgical Gastro	42	Neonatology	04
Neuro Surgery	40	Dermatology	03
Trauma Ventilator Unit	36	Medical gastroenterology	03
Respiratory Medicine	32	Radiotherapy	03
Pulmonary medicine	26	Orthopedics	03
Emergency medicine	20	Endocrinology	03
Neurology	19	Medical oncology	02
Plastic surgery	11	Community Health	02
Urology	09	Gastro	01
Surgical oncology	09	Dental	01
Nephrology	08	Otolaryngology	01
Pulmonology	01	Others	01

Among 1058 Candida isolates, 624 were from males and 434 were from females. Candida tropicalis was the most prevalent, with 340 isolates, which make up 32.13% of the total sample as mentioned in Table 3. This species represented nearly one-third of all isolates. Candida parapsilosis followed, with 145 isolates, accounting for 13.7% of the total. This species was also relatively common but far less abundant than Candida tropicalis. Candida auris had 100 isolates, which is 9.45% of the total. This species garnered increasing attention due to its multidrug resistance and emerging role as a hospital-associated pathogen. Candida albicans, traditionally the most common human pathogen in the Candida genus, had 96 isolates (9.07%), making it one of the more frequent species in this sample, though it ranks just below C. auris here. Candida utilis came in fifth, with 88 isolates (8.31%). Although it is an industrial yeast, it can also be found in clinical settings. Candida glabrata was present in 38 isolates, contributing 3.59% of the total sample. C. glabrata was often a concern in immunocompromised patients, as it can exhibit resistance to azole antifungals. The percentage of species distribution of the remaining isolates is explained in Table 3.

**Table 3:** Isolates of Candida species in bloodstream infection N = Total number of positive samples. n = Total number of isolates of Candida species.

Species	n (%) (N=1058)
Candida tropicalis	340 (32.13)
Candida parapsilosis	145 (13.7)
Candida auris	100 (9.45)
Candida albicans	96 (9.07)
Candida utilis	88 (8.31)
Candida glabrata	38 (3.59)
Candida krusei	17 (1.6)
Candida rugosa	5 (0.47)
Candida guilliermondii	4 (0.37)
Candida pelliculosa	4 (0.37)
Candida haemulonii	1 (0.09)
Candida lipolytica	1 (0.09)
Candida lusitaniae	1 (0.09)

The antibiogram report showed the susceptibility of various Candida species to different antifungal agents as mentioned in Table 4. Caspofungin showed high susceptibility across most species, e.g., Candida albicans at 94.7%, Candida parapsilosis at 98.6%, and Candida tropicalis at 97.5%. Fluconazole displayed variable effectiveness as low susceptibility was found in Candida auris (8.9%) and Candida glabrata (35.1%). Moderate effectiveness was observed in Candida albicans (77%) and Candida tropicalis (79.5%). Voriconazole maintained relatively high susceptibility, with better performance compared to fluconazole in most species, e.g., Candida albicans (84%) and Candida glabrata (78.9%). Candida species tested include Candida albicans, Candida auris, Candida glabrata, Candida krusei, Candida parapsilosis, Candida tropicalis and Candida utilis. Antifungal agents evaluated are amphotericin B, caspofungin, fluconazole, and voriconazole. For each Candida species, the number of isolates tested and their respective susceptibility rates (percentages) to the antifungals are provided.

**Table 4:** Susceptibility of Candida species to antifungal drugs n = Total number of isolates of Candida species. p-value for amphotericin B = p > 0.05 (Sensitivity to amphotericin B is not significantly different across Candida species.) p-value for caspofungin = p < 0.05 (Sensitivity to caspofungin is significantly different across Candida species.) p-value for fluconazole = p < 0.05 (Sensitivity to fluconazole is highly significantly different across Candida species.) p-value for voriconazole = p < 0.05 (Sensitivity to voriconazole is significantly different across Candida species.)

AMA	Blood					
	Candida tropicalis	Candida parapsilosis	Candida auris	Candida albicans	Candida utilis	Candida glabrata
	n=349	n=148	n=101	n=100	n=88	n=38
Amphotericin B	44/162	44/49	41/101	35/100	27/88	15/38
	(27.2%)	(89.8%)	(7.3%)	(5.7%)	(18.5%)	(39.4%)
Caspofungin	320/336	143/145	96/100	90/95	78/80	25/33
	(95.2%)	(98.6%)	(96%)	(94.7%)	(97.5%)	(75.8%)
Fluconazole	279/349	103/148	9/101	77/100	70/88	13/37
	(79.9%)	(69.6%)	(8.9%)	(77%)	(79.5%)	(35.1%)
Voriconazole	293/349	123/147	37/97	84/100	61/87	30/38
	(84%)	(83.7%)	(38.1%)	(84%)	(70.1%)	(78.9%)

The results indicate that for caspofungin, fluconazole, and voriconazole, the chi-squared values are relatively high, suggesting a statistically significant association between Candida species and sensitivity. For amphotericin B, the chi-squared value is lower, indicating a weaker association.

## Discussion

The annual distribution of six Candida species from 2020 to 2024 is shown in Table 5, detailing both the isolate counts and their respective percentages of the total Candida isolates per year; Candida tropicalis demonstrates a significant increase over time, rising from 35% in 2020 to 76.9% in 2024, while Candida parapsilosis exhibits a gradual increase, and Candida auris shows a notable upward trend, suggesting a growing clinical concern; Candida albicans remains relatively stable, Candida utilis fluctuates with a peak in 2022, and Candida glabrata shows a declining trend, highlighting the dynamic nature of Candida species prevalence over these five years.

**Table 5:** Year-wise isolation trends of Candida species N = Total number of species n = Total number of samples

CandidaSpecies	2020 N(%) n=137		2021 N(%) n=246		2022 N(%) n=261		2023 N(%) n=162		2024 N(%) n=252	
Candida tropicalis	48	(35%)	92	37.3(%)	34	13.0(%)	50	30.8(%)	194	76.9(%)
Candida parapsilosis	6	(4.3%)	26	10.5(%)	49	18.7(%)	31	19.1(%)	55	21.8(%)
Candida auris	15	(10.3%)	22	8.9(%)	21	8.0(%)	22	13.5(%)	64	25.3(%)
Candida albicans	11	(8.0%)	20	8.1(%)	22	8.4(%)	24	14.8(%)	34	13.4(%)
Candida utilis	13	(9.4%)	3	1.2(%)	45	17.2(%)	18	11.1(%)	18	7.1(%)
Candida glabrata	8	(5.8%)	8	3.2(%)	14	5.3(%)	6	3.7(%)	5	1.9(%)

Table 5 also displays the percentage distribution of different Candida species over five years, from 2020 to 2024, revealing notable shifts in their prevalence. Candida tropicalis started with a high percentage of 35% in 2020 and 37.3% in 2021, experienced a sharp decline to 13% in 2022, then increased to 30.8% in 2023, and surged dramatically to 76.9% in 2024, suggesting a potential outbreak or a significant change in its prevalence. Candida parapsilosis showed a consistent upward trend, rising from 4.3% in 2020 to 21.8% in 2024, indicating its growing presence. Candida auris remained relatively stable in the first three years, around 8-10%, but increased to 13.5% in 2023 and then significantly to 25.3% in 2024, raising concerns due to its association with healthcare-associated infections and drug resistance. Candida albicans fluctuated, starting at 8% and reaching 14.8% in 2023 before decreasing slightly to 13.4% in 2024, suggesting variability in its prevalence. Candida utilis showed the most erratic pattern, dropping from 9.4% to 1.2% and then rising to 17.2% before declining to 7.1%, indicating possible variations in sampling or environmental factors. Finally, Candida glabrata showed a general decline from 5.8% in 2020 to 1.9% in 2024, suggesting a decreasing prevalence over time. Overall, the data reveals dynamic changes in the distribution of Candida species, with notable increases in Candida tropicalis, Candida parapsilosis, and Candida auris, warranting further investigation to understand the underlying causes and potential implications for public health [16].

Table 4 meticulously details the antifungal susceptibility of six Candida species (C. tropicalis, parapsilosis, auris, albicans, utilis, and glabrata) isolated from blood samples, against four common antifungal drugs (amphotericin B, caspofungin, fluconazole, and voriconazole). It presents the raw count of susceptible isolates out of the total tested for each drug-species combination, alongside the corresponding percentage of susceptibility, providing a clear picture of each drug's effectiveness against different Candida species; notably, caspofungin demonstrates consistently high susceptibility across all species, while amphotericin B shows variable efficacy, particularly low against auris and albicans; fluconazole and voriconazole also exhibit varied susceptibility profiles, with auris showing notably low susceptibility to both, suggesting potential resistance patterns that warrant clinical attention. This data is crucial for informing treatment decisions and understanding the evolving landscape of antifungal resistance in clinically significant Candida species. 

The use of invasive medical devices such as central venous catheters, artificial joints, or endotracheal tubes increases the risk of candidemia. These devices can provide a direct pathway for Candida to enter the bloodstream, especially in hospital settings where these devices are commonly used for patients undergoing treatments or surgeries. The longer a device is in place, the higher the risk of infection [17]. The increasing cases of candidemia in tertiary care hospitals in India reflect broader trends in India’s healthcare system [18]. The rise in Candida bloodstream infections in these prestigious institutions is a result of a combination of patient factors, healthcare practices, and regional challenges [19]. Here's a detailed analysis of the key contributing factors in these hospitals: prevalence of immunocompromised patients, prolonged use of invasive medical devices, antibiotic overuse and resistance, high patient load and crowding in hospitals, regional challenges and environmental factors, improvements in diagnostics and surveillance, and antifungal resistance and limited therapeutic options [20,21]. Some patients, particularly those with underlying conditions such as diabetes or chronic kidney disease, can develop chronic or recurrent Candida infections. These patients are at risk of persistent or recurrent episodes of superficial infections (e.g., vaginal candidiasis, oral thrush) or deep-seated infections, which can become difficult to treat and may require prolonged antifungal therapy [22].

Limitations

This study, while providing valuable insights into Candida species prevalence and antifungal resistance, has several limitations. Its cross-sectional design restricts the ability to establish temporal relationships and track resistance progression over time. Being a single-center study in North India, the findings may not be generalizable to other populations or settings. The retrospective data collection from laboratory records introduces potential biases from incomplete or inaccurate data. Selection bias is also a concern as only clinically relevant isolates with susceptibility testing were included, potentially excluding other cases. The focus on bloodstream infections limits the understanding of Candida infections at other sites. Furthermore, limited demographic information hinders the exploration of risk factors, and potential misclassification due to reliance on laboratory records could affect accuracy. The lack of clinical correlation restricts the translation of findings into practice, and data entry errors from using Microsoft Excel are possible. These limitations should be considered when interpreting the study's results.

## Conclusions

Bloodstream infections in India present a significant public health challenge, with a notable burden on both individual health and healthcare resources. At one time, Candida albicans was the most common organism but now Candida auris is the most commonly resistant pathogen in all the hospitals in India and it is increasing resistance continuously. Addressing underlying risk factors, such as diabetes, and implementing effective infection control measures in healthcare settings are crucial steps in reducing the burden of Candida infections in India. Candida resistance is a growing clinical challenge that requires urgent attention. Risk factors such as immunocompromised patients, indwelling devices, and excessive antifungal use contribute to the rise of resistant strains. Surveillance and diagnostic challenges remain barriers to timely and accurate treatment, and effective prevention strategies, including antifungal stewardship and infection control, are vital. Moving forward, the development of new antifungal agents, rapid diagnostic tests, and personalized treatment strategies offer hope in addressing the threat of Candida resistance.
